# Interferon Gamma Suppresses Collagen-Induced Arthritis by Regulation of Th17 through the Induction of Indoleamine-2,3-Deoxygenase

**DOI:** 10.1371/journal.pone.0060900

**Published:** 2013-04-16

**Authors:** Jaeseon Lee, Jennifer Lee, Mi-Kyung Park, Mi-Ae Lim, Eun-Mi Park, Eun-kyung Kim, Eun-Ji Yang, Seon-Yeong Lee, Joo-Yeon Jhun, Sung-Hwan Park, Ho-Youn Kim, Mi-La Cho

**Affiliations:** The Rheumatism Research Center, Catholic Research Institute of Medical Science, The Catholic University of Korea, Seoul, South Korea; University Hospital of Heidelberg, Germany

## Abstract

C57BL/6 mice are known to be resistant to the development of collagen-induced arthritis (CIA). However, they show a severe arthritic phenotype when the *Ifng* gene is deleted. Although it has been proposed that IFN-γ suppresses inflammation in CIA via suppressing Th17 which is involved in the pathogenesis of CIA, the exact molecular mechanism of the Th17 regulation by IFN-γ is poorly understood. This study was conducted to 1) clarify that arthritogenic condition of IFN-γ knockout (KO) mice is dependent on the disinhibition of Th17 and 2) demonstrate that IFN-γ-induced indoleamine2,3dioxgenase (IDO) is engaged in the regulation of Th17. The results showed that the IFN-γ KO mice displayed increased levels of IL-17 producing T cells and the exacerbation of arthritis. Also, production of IL-17 by the splenocytes of the IFN-γ KO mice was increased when cultured with type II collagen. When *Il17* was deleted from the IFN-γ KO mice, only mild arthritis developed without any progression of the arthritis score. The proportion of CD44^high^CD62L^low^ memory-like T cells were elevated in the spleen, draining lymph node and mesenteric lymph node of IFN-γ KO CIA mice. Meanwhile, CD44^low^CD62L^high^ naïve T cells were increased in IFN-γ and IL-17 double KO CIA mice. When Th17 polarized CD4+ T cells of IFN-γ KO mice were co-cultured with their own antigen presenting cells (APCs), a greater increase in IL-17 production was observed than in co-culture of the cells from wild type mice. In contrast, when APCs from IFN-γ KO mice were pretreated with IFN-γ, there was a significant reduction in IL-17 in the co-culture system. Of note, pretreatment of 1-methyl-DL- tryptophan, a specific inhibitor of IDO, abolished the inhibitory effects of IFN-γ. Given that IFN-γ is a potent inducer of IDO in APCs, these results suggest that IDO is involved in the regulation of IL-17 by IFN-γ.

## Introduction

Rheumatoid arthritis (RA) is an autoimmune disease that is characterized by multiple, persistent synovial inflammation that can lead to joint destruction if left untreated. Activated by unidentified autoantigens, T cells are known to play a crucial role in initiating and maintaining the inflammatory process. Collagen-induced arthritis (CIA) model in DBA/1 mice has been widely used as an animal model of RA. In this model, T helper 1(Th1) immune response was thought to be involved in the pro-inflammatory process. However, the role of IFN-γ, a characteristic Th1 cytokine, was of interest as IFN-γ receptor deficient mice showed rather exacerbated arthritic phenotypes of CIA [1]. This was further supported by the report that C57BL/6 mice, which are known to be resistant to the development of CIA, developed arthritis with severe inflammation when the *Ifng* gene was deleted [2]. Moreover, the protective effect of IFN-γ is not limited to CIA. It has been reported that a deficiency in IFN-γ may contribute to pro-inflammatory conditions such as experimental autoimmune neuritis [3], experimental autoimmune encephalomyelitis [4], delayed-type hypersensitivity [5], and acute graft-versus-host disease [6].

The inhibitory function of IFN-γ was once suggested to be associated with IL-1β. It was reported that the expression of IL-1β increased and arthritis was ameliorated with anti-IL-1β treatment in IFN-γ deficient C57BL/6 mice [7]. The authors argued that IFN-γ was a negative regulator in the development of autoimmune arthritis in B6 mice and in part was involved with the control of IL-1β production. However, with the emergence of the Th17 cells which are crucial in autoimmune diseases, the protective role of IFN-γ has been explained to be associated with the suppression of Th17 and its effector function. It was reported that IFN-γ could suppress the IL-23-driven Th17 development [8]. And the exacerbated CIA in IFN-γ deficient mice was demonstrated to be due to lack of Th17 inhibition [9]. Nevertheless, very little is known about the exact mechanism of how IFN-γ exerts its inhibitory effect on Th17. It has been proposed that the inhibition of IL-17 by IFN-γ is dependent on STAT-1 [10], but there are no details on its mechanism.

Indoleamine 2,3dioxgenase (IDO) is a catabolic enzyme which mediates tryptophan degradation [11]. It is widely expressed in various cells including tumor cells [12], dendritic cells [13], macrophages [14], microglia [15], eosinophils [16], fibroblasts [17], and endothelial cells [18]. IDO expression is known to be induced by various stimuli including cytokines such as IFN-γ, pathogen-associated molecular patterns and co-stimulatory molecules [19]. In the context of immune cells, the role of IDO is to suppress T cell generation by depriving tryptophan and producing the toxic tryptophan metabolite-kynurenine [20]. Of note, it has been demonstrated to play a crucial role in the suppression of autoimmunity as it regulates autoreactive T cells [21]. Specifically, several studies demonstrated that IDO negatively regulates the Th17 response [22], [23]. Therefore, we hypothesized that IDO induced by IFN-γ is involved in the suppression of Th17 in CIA. The present study demonstrated that the suppression of Th17 by IFN-γ was abolished with 1-methyl-DL- tryptophan, a specific inhibitor of IDO.

## Materials and Methods

### Mice

Male C57BL/6, IFN-γ KO, IDO KO mice (8∼10 weeks) were purchased from Jackson laboratory. IL-17 KO mice were obtained from Dr. Y. Iwakura (University of Tokyo, Tokyo, Japan). IFN-γ KO mice were backcrossed to IL-17 KO mice over 10^th^generations and double KO (DKO) mice were selected for by PCR, which were given standard mouse chow (Ralston Purina) and water *ad libitum*. All experimental procedures were examined and approved by the Animal Research Ethics Committee of the Catholic University (Seoul, Korea).

### Induction and Assessment of Arthritis

The protocol to develop arthritis of the C57BL/6 mice was referred to Julia J Inglis et al [24]. Briefly, 200 µg/ml chicken type II collagen (CII, Chondrex, Seattle, WA) emulsified with CFA (Arthrogen-CIA, Redmond, WA) (1∶1w/v) was injected at the base of tail and in a slightly more anterior site of wild type and IFN-γ KO mice. Three weeks later, a boost mixed with CII (100 µg/ml) emulsified with IFA (Difco, Detroit, MI) (1∶1w/v) was given with the same protocol as above. Arthritic score measurements were performed as follows: 0 = no joint swelling; 1 = slight edema and erythema limited to the foot or ankle; 2 = slight edema and erythema from the ankle to the tarsal bone; 3 = moderate edema and erythema from the ankle to the tarsal bone; and 4 = edema and erythema extending from the ankle to the entire leg, with severe swelling of the wrist or ankle. The final arthritis score was calculated as the sum of scores from all four legs, which were assessed by three independent observers with no knowledge of the experimental groups.

### Preparation of Cell Suspension

Spleens were removed from IFN-γ KO or IFN-γandIL-17 DKO mice 5 weeks after primary immunization. Spleen tissue was minced. Splenic red blood cells were removed with an ACK lysis buffer (2.06% Tris, pH 7.65, and 0.83% NH_4_Cl). Cell suspension was passed through a 40 µm strainer (BD Falcon, Bedford, MA) and re-suspended with 5% fetal bovine serum (Gibco, Grand Island, NY) containing RPMI1640 (Gibco) media.

### CD4^+^ T cell Isolation and Stimulation

Spleen cells were washed with 0.5% bovine serum albumin (BSA, Sigma, St Louis, MO), 5 mM ethylenediaminetetraacetic acid (EDTA, Sigma) containing PBS buffer (pH7.2). After centrifugation at 1300 rpm and 4°C, cells were incubated with CD4-coated magnetic beads (MiltenyiBiotec, BergischGladbach, Germany) and isolated on MACS separation columns (MiltenyiBiotec). Positively selected CD4^+^ T cells were stimulated with plate-bound 0.5 µg/ml of anti-CD3 mAbs (BD biosciences, San Jose, CA), soluble 1 µg/ml of anti-CD28 mAbs (BD biosciences), 2 µg/ml of anti-IFN-r Abs (R&D systems, Minneapolis, MN), 2 µg/ml of anti-IL-4 Abs (R&D systems), 2 ng/ml of recombinant TGF-β (R&D systems), 20 ng/ml of recombinant IL-6 (R&D systems) for 3 days for Th17 polarization. Negatively selected non-CD4 cells were regarded as antigen presenting cells (APCs) and stimulated with 100 ng/ml of recombinant IFN-γ for 3 days with or without pretreatment with 100 µM of 1 methyl-DL-tryptophan (Sigma) for 2 hours. In co-cultures using Th17 polarized cell and APC^Nil^/APC^IFN−r^, both cells were harvested, washed and concentrated with 5×10^5^ cells/well in a 24well plate. APCs were irradiated at 3000 rad before the co-culture. Each culture supernatant was collected and used for cytokine ELISA.

### Histology

Mouse joint tissue was fixed in 4% paraformaldehyde, decalcifiedin EDTA bone decalcifier and embedded in paraffin. The sections (7 µm) were stained with hematoxylin and eosin, Safranin O, and toluidine blue to detect proteoglycans. For immunohistochemistry, mouse joint or spleen tissue sections were blocked with 1% normal goat serum followed by stained with antibodies to IL-17 (1 µg/ml, SantaCruz, CA, USA), RANKL (200 ng/ml, SantaCruz), RANK (200 ng/ml, SantaCruz), IL-2 (100 ng/ml, SantaCruz), IL-15 (200 ng/ml, SantaCruz), IL-21 (200 ng/ml, SantaCruz), and isotype control antibody (1 µg/ml, SantaCruz). The sections were then incubated with the appropriate biotinylated secondary antibodies (SantaCruz) and followed by an avidin–enzyme complex. PBS containing 0.05% Tween 20 was used for washing after each step. Chromogenic reactions were visualized with 3,3′-diaminobenzidine (Sigma), and the nuclei were counterstained with hematoxylin. Slides were mounted in permanent mounting media (Dako, Glostrup, Denmark).

### Mixed Lymphocyte Culture

Splenocytes (2×10^5^ cells/well) were plated on 96well-flat-bottomed plate in 200 µl/well. They were stimulated with CII 20 µg/ml or anti-CD3 0.5 µg/ml for 4 days followed by the incorporation of 1 µCi/ml [3H]-thymidine (GE Healthcare, Piscataway, NJ) for the last 18 hours of the indicated total culture interval. Then the radioactivity was measured with a Micro Beta (Pharmacia Biotech, Piscataway, NJ).

### Analysis of Anti-CII IgG Antibody

Blood samples collected from the mice were assayed using mouse IgG/IgG1 ELISA quantification kit (Bethyl lab, Montgomery, TX). Briefly, CII (4 µg/ml) was coated in PBS at 4°C overnight. Levels of IgG1 and IgG2a were measured in mice sera diluted 1000-fold, according to the manufacturer’s instruction.

### Quantitative RT-PCR

Messenger RNA was isolated using Trizol (Invitrogen, Grand Island, USA) according to the manufacturer’s instructions. Total RNA (2 µg) was reverse transcribed at 10 min for 25°C, 30 min for 55°C and finally 5 min for 85°C using Transcriptor First Strand cDNA Synthesis kit (Roche, Mannheim, Germany). Real-time PCR amplification was performed with 0.3–0.5 µl reverse transcription product in a LightCycler1.5 (Roche) by using FastStart Universal SYBR Green Master (Roche) according to the manufacturer’s guidelines. The following sense and antisense primers for each molecules were used for:IL-17, 5′-GGTCAACCTCAAAGTCTTTAACTT-3′ (sense) and 5′-TTAAAAATGCAAGTAAGTTTG-3′ (antisense);β-actin, 5′-GAAATCGTGCGTGACATCAAAG-3′ (sense) and 5′-TGTAGTTTCATGGATGCCACAG-3′ (antisense).The PCR cycling conditions were as follows: 10 min at 95°C, 45cycles of 15 s at 95°C, 45 s at 60°C, and 20 s at 72°C. To verify that equivalent amounts of RNA were added to each PCR reaction, PCR amplification of the murine β-actin was performed for each sample. Relative fold induction was calculated using the equation 2^−(ΔΔCp)^, where ΔΔCp is ΔCp_(stimulated)_− ΔCp_(control)_, ΔCp is Cp_(IL17)_− Cp_(β-actin)_, and Cp is the cycle at which the threshold is crossed. PCR product quality was monitored using post-PCR melting curve analysis.

### Flowcytometry

Single cell suspensions of spleen were washed with FACS buffer (0.5% BSA, 0.02 N sodiumazide in PBS, pH 7.4) and stained with following antibodies:APC conjugated anti CD44 antibody, PE conjugated anti CD62L antibody (BD biosciences), PerCP conjugated anti CD4 antibody (eBiosciences). For intracellular FACS staining, cells were fixed with Cytofix/cytoperm solution (BD) followed by washing with permeablization buffer (BD) and then stained with PE anti mouse IL-17 antibody (ebioscience). FACS analysis was performed using a FACS Calibur (BD, San Diego, CA), and the data were analyzed with Flow Jo software, version 7.6 (Treestar, Ashland,OR). The lymphocyte group was gated on the whole cell region by the forward/side scatter properties, CD4^+^ region was gated on lymphocyte followed by fluorescence only for the analysis of naïve/memory T cell population.

### Detection of IL-17 by ELISA

Antibodies directed against mouse IL-17 and biotinylated anti-mouse IL-17 Abs (R&D Systems) were used as the capture and detection Abs, respectively. The fluorescent substrate HRP-avidin (R&D Systems) was used for color development. The amounts of cytokines present in the test samples were determined from standard curves established with serial dilutions of recombinant IL-17 (R&D Systems).

### Statistics

Experimental values are presented as mean ± SEM of triplicate cultures and representative of experiments performed on three occasions. Statistical significance was determined by Mann-Whitney U test or ANOVA with Bonferroni’s post-hoc test using the Graphpad Prism (v.5.01). Values of p<0.05 were considered statistically significant. *, p<0.05; **, p<0.01; ***, p<0.001.

## Results

### IFN-γ Deficient Mice Demonstrated Increased IL-17 Production and Severe Phenotype of Collagen-induced Arthritis

**Figure 1:**
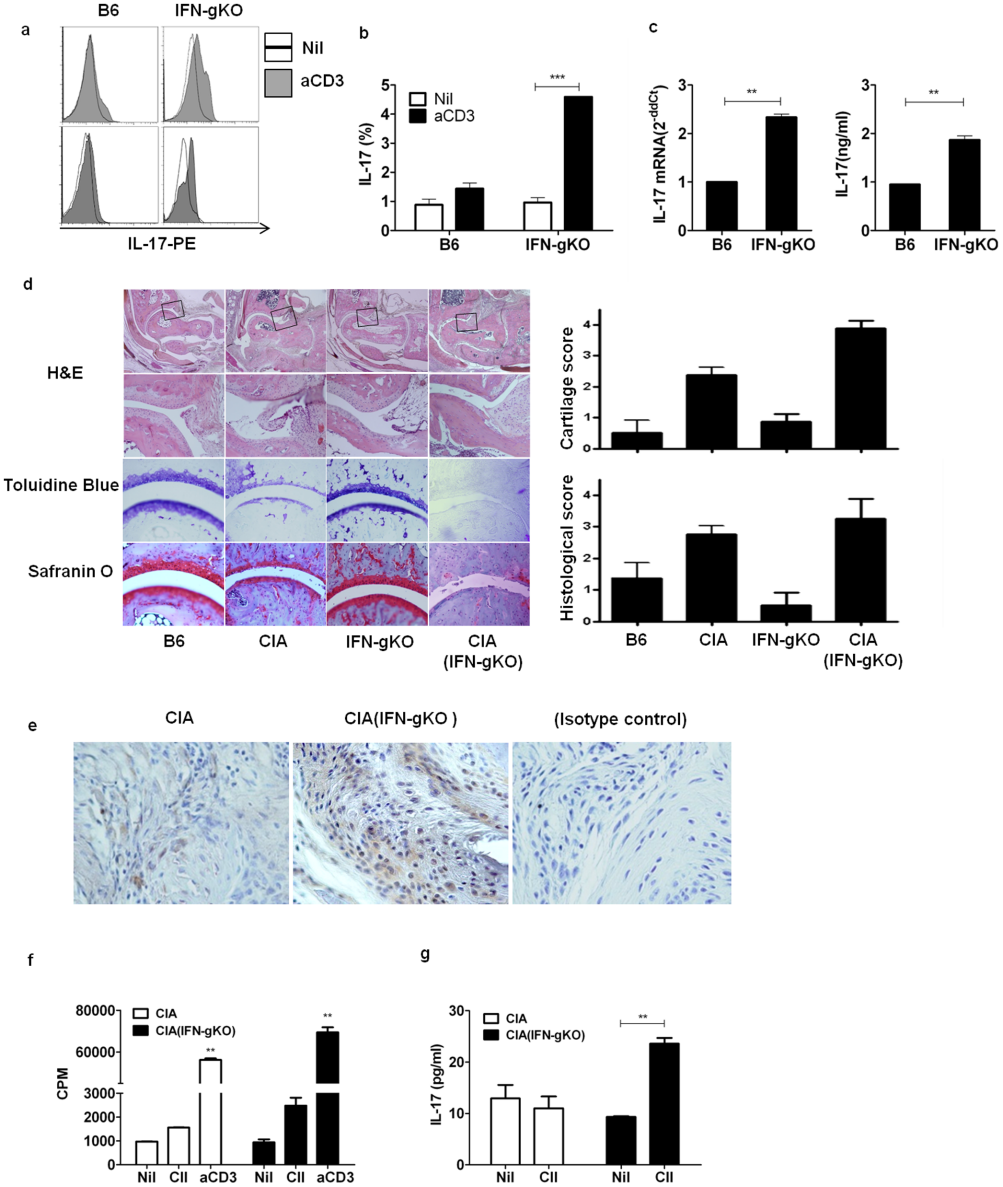
IFN-γ deficient mice demonstrated increased IL-17 production and severe phenotype of collagen-induced arthritis (CIA). Splenocytes from WT B6 or IFN-γ KO B6 mice were cultured with media alone and with 0.5 µg/ml of plate bounded-anti CD3 monoclonal antibodies for 48 hrs (a) to 72 hrs (b) and stained with PE conjugated anti-IL-17 antibody. The proportion of IL-17 producing cells was measured using flow cytometry. (c) Splenocytes from WT B6 or IFN-γ KO B6 mice were cultured in a Th17 polarizing condition. The mRNA expression of IL-17 and the protein level of IL-17 in the supernatant were measured. (d) Sections from the hind paws of WT B6 with or without CIA and IFN-γ KO B6 with or without CIA were obtained at 9^th^ week after primary CII+CFA immunization. Joint tissue sections were stained with Hematoxylin and Eosin, Toluidine Blue, Safranin O. Infiltration of immune cells in joint synovium between tibia and tarsal bone was observed using microscope and scored according to the referred standard. (e) IL-17 was significantly high in CIA (IFN-γ KO). Joint sections were fixed with paraffin to perform Immunohistochemistry. Original magnification, 400x. (f) Splenocytes from WT B6 with CIA and IFN-γ KO B6 mice were cultured with media alone, 50 µg/ml of CII, 0.5 µg/ml of plate-bounded anti CD3 mAb for 96 hrs, then cell proliferative responses were determined by ^3^H-thymidine incorporation assay. Data is represented as the mean counts per minute (CPM). (g) Splenocytes from WT B6 with CIA and IFN-γ KO B6 with CIA were stimulated with 50 µg/ml of CII for 72 hrs. The level of IL-17 in the culture supernatant was measured using ELISA. Mann-Whitney U test (b,c,g) and ANOVA with post hoc analysis (d,f) was used. Values are presented as the mean ± standard deviation of three independent experiments. *, p<0.05, **, p<0.005 ***, p<0.001.

As previous studies have reported the suppression of IL-17 production by IFN-γ, the results showed that the proportion of IL-17 producing cells among total splenocytes was significantly higher in IFN-γ KO mice than in WT B6 KO mice when stimulated with anti CD3 antibody (Figure 1a, 1b). Then, CD4+ cells were isolated and cultured in Th17 polarizing conditions. Th17 polarized cells from IFN-γ KO mice produced more IL-17 than those from WT mice, which was addressed by measuring IL-17 mRNA expression and the concentration of IL-17 in the supernatant (Figure 1c).When CIA was induced, the joint destruction was more pronounced in IFN-γ KO mice (Figure 1d). IL-17 producing cells were also more intensely infiltrated in the joints of CIA induced IFN- γ KO mice (Figure 1e). As well, there was an increased cell proliferation in response to CII and anti CD3 antibody stimulation in IFN- γ KO mice when compared to the WT b6 KO mice (CIA nil: 973±4.2, CIA CII: 1558±14.1, CIA aCD3∶56232±1023.9; CIA (IFN-γ KO) nil: 941.5±177.5, CIA (IFN- γ KO) CII: 2481±473.8, CIA (IFN- γ KO) aCD3∶69423±3438.0; CPM) (Figure 1f). CII-specific IL-17 production was observed only in the splenocytes from IFN-γ KO mice (CIA nil: 12.9±4.6 pg/ml, CIA CII: 11.0±4.0 pg/ml, CIA (IFN- γ KO): 9.3±0.3 pg/ml, CIA (IFN- γ KO): 23.6±2.0 pg/ml) (Figure 1g).

### IFN-γ and IL-17 Double Knockout Mice did not Develop Progressive Arthritis

**Figure 2:**
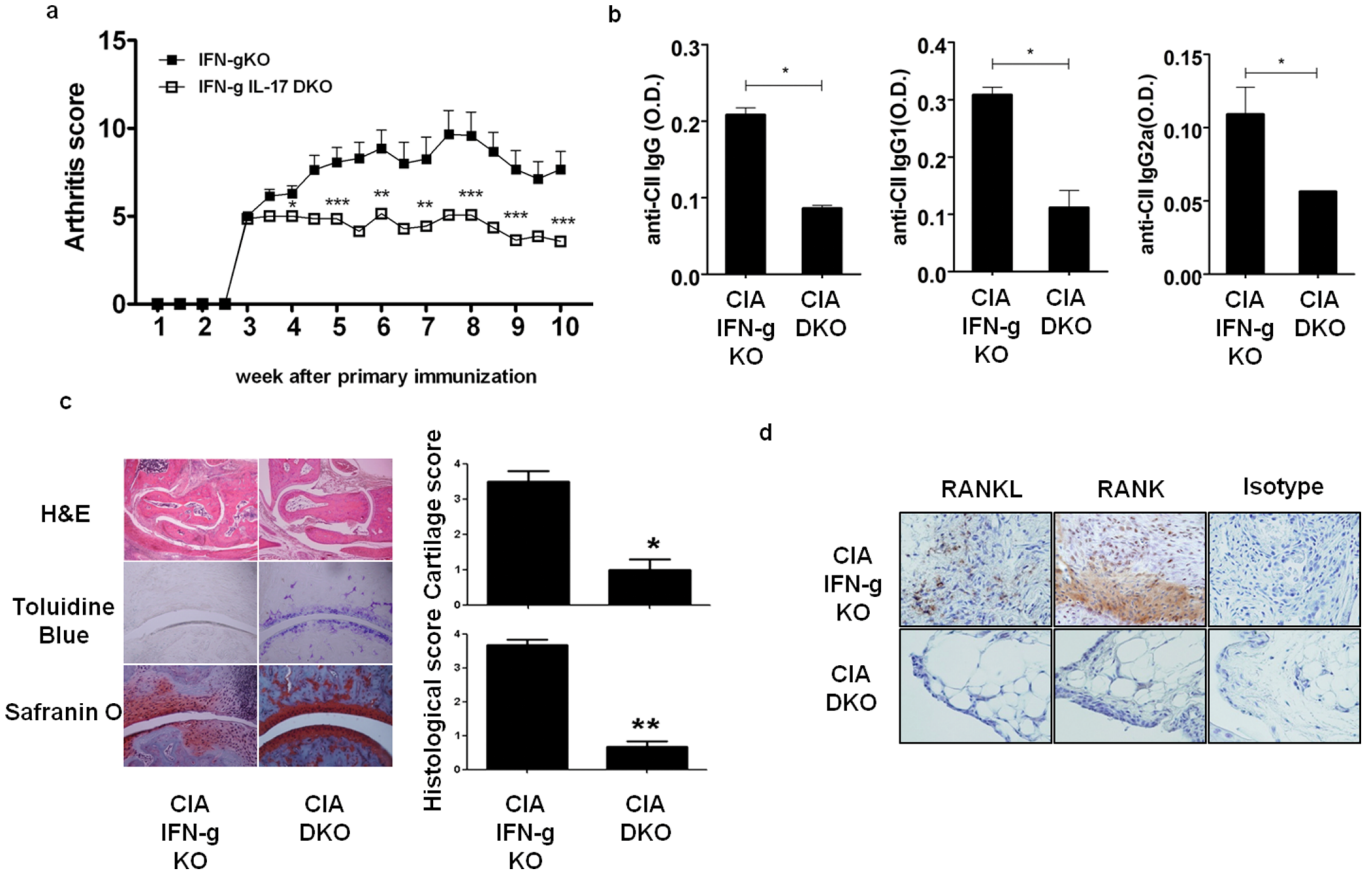
IFN-γ/IL-17 double knockout mice did not develop arthritis. (a) Comparison of arthritis score during 10 weeks after 1^st^ immunization. (b) Serum was collected at 5^th^ week after CIA induction, the level of anti CII IgG, IgG1, IgG2 was determined by ELISA. (c) Joint histology of CIA (IFN-γ KO, n = 14) and CIA (IFN-γIL-17 DKO, n = 14) at 10^th^ week after 1^st^ immunization. Joint tissue sections were stained with Hematoxylin and Eosin, Toluidine Blue, Safranin O. (d) Immunohistochemistry. RANKL or RANK positive cells were not seen in CIA (IFN-γ/IL-17 DKO) joint section. Original magnification,200x. Mann-Whitney U test (b) used. Values are presented as the mean ± standard deviation. *, p<0.05, **, p<0.005 ***, p<0.001.

To demonstrate that IL-17 was the major cytokine that lead to the exacerbation of arthritis in IFN-γ deficient mice, CIA was induced in IFN-γ IL-17 DKO mice. Until 10 weeks after immunization, only mild CIA without progression (Arthritis score 3) was developed in DKO mice while IFN-γ KO mice exhibited severe joint swelling (Figure 2a). In addition, the level of anti CII IgG, IgG1 measured in the sera obtained at 5 weeks post-immunization was significantly lower in DKO mice (anti CII IgG, CIA(IFN-γ KO): 0.208±0.018, CIA(IFN-γIL-17 DKO): 0.086±0.008; anti CII IgG1, CIA(IFN-γ KO): 0.308±0.027, CIA(IFN-γ IL-17 DKO): 0.112±0.060; anti CII IgG2a, CIA(IFN-γ KO): 0.109±0.037, CIA(IFN-γ IL-17 DKO): 0.056±0.001 O.D.(450 nm)) (Figure 2b).Mice were sacrificed at 10 weeks post-immunization to evaluate the histology of the joints. Joint destruction was not observed in the joints of DKO mice (Histology score, CIA(IFN-γ KO): 3.67±0.289, CIA(IFN-γ IL-17 DKO): 0.67±0.289; Cartilage score, CIA(IFN-γ KO): 3.50±0.500, CIA(IFN-γ IL-17 DKO): 1.00±0.500) (Figure 2c). RANK and RANKL expression in the synovium was also addressed as RANK-RANKL interaction has been known to be essential in the differentiation of osteoclast which plays the key role in bone destruction in CIA. As expected, the expression of RANKL, RANK was low in the synovium of CIA-induced DKO mice (Figure 2d).Collectively, these data suggest that IL-17 is critical in collagen-specific inflammation in IFN-γ KO mice.

### Memory Type CD4+ T cells were Decreased in IFN-γ IL-17 DKO CIA Mice

**Figure 3:**
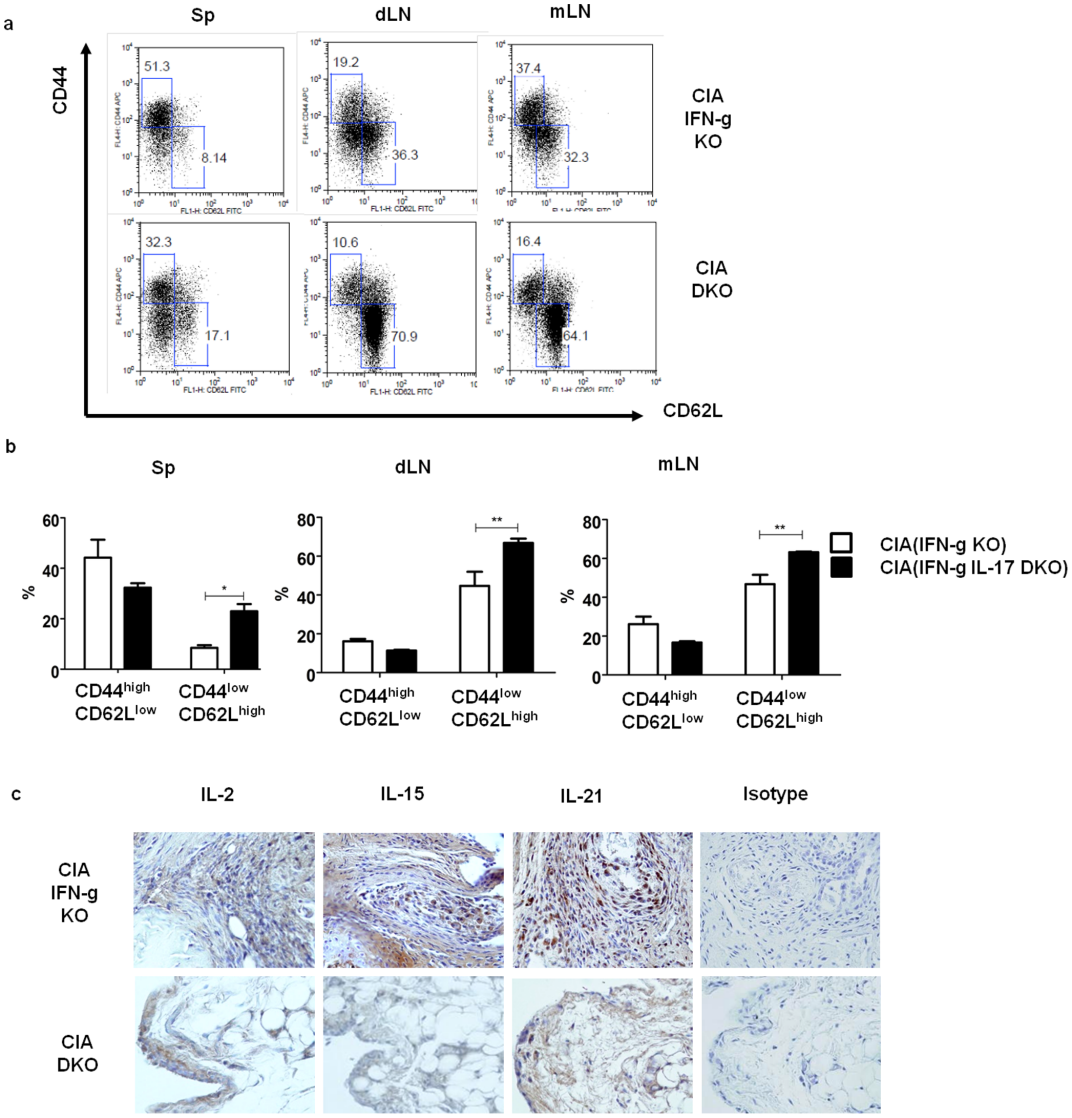
The proportion of naïve T cells increased in IFN-γ/IL-17 double knockout mice compared to that of IFN-γ KO mice. Spleen, draining lymph nodes and mesenteric lymph node were removed at 5^th^ week after 1^st^ immunization, and prepared for single cell suspension. They were stained with PerCP - anti CD4 ab, APC - anti CD44 ab, and FITC - anti CD62L ab for flowcytometry. (a) Data represents one of three experiments. (b) CD4^+^CD44^high^CD62L^low^ (memoryCD4^+^T cells) T cell population was decreased in CIA (IFN-γIL-17 DKO) group while CD4^+^CD44^low^CD62L^high^ (naïve CD4^+^ T cells) population was increased. (c) IL-2, IL-15 and IL-21 were strongly expressed in the joint synovium of CIA (IFN-γ KO). Original magnification, 200x. Mann-Whitney U test used (b). Values are presented as the mean ± standard deviation of four independent experiments. *, p<0.05, **, p<0.005 ***, p<0.001.

It can be easily be deduced that the population of antigen specific memory T cell is decreased in DKO mice as severe arthritis is not developed in DKO mice. Indeed, the proportion of memory type CD44^low^CD62L^high^ CD4+ T cells were decreased in DKO mice. On the contrary, the proportion of naïve CD4+ T cells significantly increased in DKO mice compared to WT mice at 5 weeks post-immunization (Figure 3a, 3b). To further explain the altered subpopulation of CD4+ T cells, the expression of the cytokines associated with T cell proliferation and memory cell development such as IL-2, IL-15 and IL-21 in the joint synovium was investigated. While IL-2 and IL-21 expression was observed in both models, there was a significant reduction in IL-15 expression within the synovium of CIA-induced DKO mice (Figure 3c).

### Suppression of IL-17 by IFN- γ is Associated with IDO

**Figure 4:**
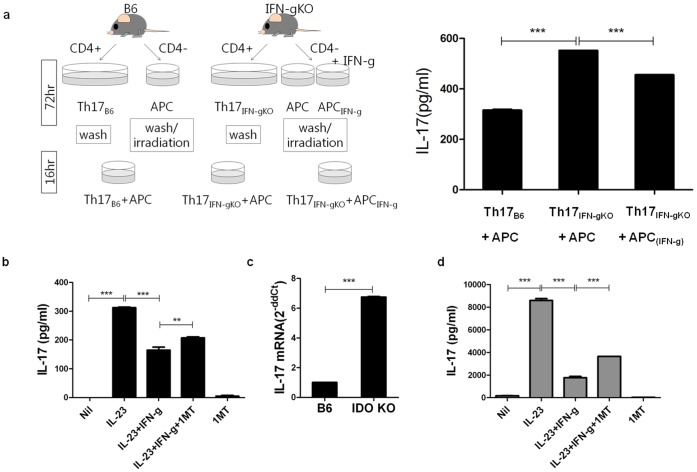
Suppression of IL-17 by IFN- γ is associated with IDO. (a) CD4^+^ T cells isolated from wild type (WT) C57BL/6 (B6) mice and IFN-γ knock out (KO) B6 mice. The cells were cultured in Th17-polarizing conditions (anti CD3 mAb, anti CD28 mAb, anti IFN-γAb, anti IL-4 Ab, TGF-β, IL-6). Non-CD4 cells were cultured with media only or 100 ng/ml of recombinant IFN-γ. After 72hours, cells were washed and only APCs were irradiated at 3000 rad. After Th17 cells and APC/APC_IFN-γ_ were co-cultured (1∶1) for 16 hrs, the culture supernatant was measured for IL-17 ELISA (right). (b) Splenocytes derived from IFN-γ KO mice were cultured solely or with combined condition with 10 ng/ml of IL-23, 50 ng/ml of IFN-γ, 100 µM 1-MT for 72 hrs. IFN-γ and 1-MT was pretreated. (c) IL-17 mRNA expression of Th17 polarized cells derived from B6 and IDO KO mice splenocyte. (d) Splenocytes derived from IL-1Ra KO mice were cultured solely or with combined condition with 10 ng/ml of IL-23, 50 ng/ml of IFN-γ, 100 µM 1-MT for 72 hrs. IFN-γ and 1-MT was pretreated. ANOVA with post hoc analysis (d,f) was used. Values are presented as the mean ± standard deviation of three independent experiments. *, p<0.05, **, p<0.005 ***, p<0.001.

CD4+ T cells isolated from WT B6 and IFN- γ KO B6 were cultured in Th17 polarizing conditions. Non-CD4+ cells were cultured with media only or 100 ng/ml of recombinant IFN-γ. Th17 polarized cells and non-CD4+ cells/non-CD4+_IFN-r_cells were then co-cultured in a ratio of 1∶1. The expression of IL-17 in the co-culture supernatant was significantly low when Th17 polarized cells were co-cultured with IFN-γ treated non-CD4 cells (Figure 4a). Therefore, we hypothesized a certain molecule which was induced by IFN-γ in non CD4+ cells suppressed the production of IL-17 of CD4+ T cells. IDO was one of the candidate molecules as it was induced by IFN-γ [25] in non CD4+ cells and known to be associated with Treg [26]–[29] which suppresses Th17. Pretreatment of 1-MT partially restored the production of IL-17 suppressed by IFN-γ (Figure 4b). Furthermore, CD4+ T cells isolated from IDO KO mice produced more IL-17 than those from WT B6 mice when cultured in Th17 polarizing conditions(Figure 4c, mRNA fold increase, B6∶1.0, IDO−/−: 6.75±0.14). Next it was investigated whether IFN-γ-induced IDO exerted the same inhibitory effects in spontaneous arthritis model IL-1Ra KO mice. As expected, IL-17 production from the splenocytes of IL-1Ra KO mice decreased after IFN-γ treatment and the inhibitory effect was abolished with pretreatment with 1-MT, as in IFN-γ KO mice (Figure 4d).

## Discussion

IFN-γ is a characteristic Th1 cytokine which was believed to play a pro-inflammatory role in autoimmune arthritis model in the past. However, recent evidence has suggested that the net effect of IFN-γ is rather associated with amelioration of arthritis [30]. Indeed, Lemmel et al. showed that low dose IFN-γ treatment had therapeutic potential in human RA, although the authors did not suggest the underlying mechanism [31]. Consistent with these findings, our study demonstrated that CIA is exacerbated in IFN-γ KO mice. IFN-γ has been reported to exert anti-inflammatory effects by various mechanisms including the suppression of differentiating monocyte/macrophages in osteoclasts, which are crucial in the bone erosion process of arthritis [32]. IFN-γ also inhibits IL-1b-mediated production of MMP-1 and MMP-3 by synovial fibroblast in the early phase of arthritis [33]. Moreover, it has been reported that IFN-γ is able to facilitate regulatory T cell development [34], and most importantly inhibit the development of Th17 cells and suppress IL-17 which is a key participant in autoimmune diseases [35].

There have been several studies that investigated the contribution of IL-17 to CIA exacerbation in IFN-γ KO mice, where IL-17 neutralizing antibodies were used for blockade [36]. Kelchtermans et al. showed the amelioration of arthritis with the treatment of the IL-17 neutralizing antibody in IFN-γ KO C57BL/6 mice [10]. They also demonstrated that the level of anti CII IgG2a was significantly reduced after treatment with the IL-17 antibody. In the present study, IFN-γ and IL-17 DKO mice were used to illustrate that the proinflammatory condition driven by IFN-γ deletion largely depends on the function of IL-17. As expected, DKO mice were not susceptible to CIA, which was confirmed by comparing clinical arthritis scores, as well as histologic arthritis scores between the IFN-γ KO mice and the IFN-γ IL-17 DKO mice. The production of IgG, IgG1, IgG2a subtypes of type II collagen specific antibodies were also reduced in DKO mice. This seemed to be due to the lack of IL-17-driven antibody production. In addition, decreased expression of RANKL and RANK in the synovial tissue of DKO mice was observed. Taken together, it is clear that the suppression of the IL-17 effector function is fundamental for IFN-γ to exert its protective effect.

During the investigation of evaluating the proportion of the T cell subtype in IFN-γ KO mice and IFN-γ IL-17 DKO mice, results showed a decrease in the proportion of CD44^high^CD62L^low^ memory-like T cells in double KO mice. In contrast, CD44^low^CD62^high^naïve T cells were less frequently observed in IFN- γ KO mice. These findings were similar to the findings of Yang et al. which showed decreased proportion of memory T cells in IL-2 and STAT3 double KO mice, demonstrating the requirement of STAT3 in IL-2 deficiency-driven inflammatory conditions [37]. It is plausible to assume that in IFN-γ KO mice, naïve T cells are activated and differentiate into effector cells, which result in synovial inflammation, whereas DKO mice display suppression in inflammation where a larger proportion of naïve cells remain inactivated. Furthermore, the expression of IL-15, an important cytokine in maintaining memory T cells was diminished within the synovial tissue of the DKO mice. This seemed to have contributed to the decrease of the memory cell proportion. Further investigation is required to interpret the exact meaning of the decreased memory- like CD4+ T cells in DKO mice.

The precise molecular mechanism of IFN-γ in the suppression of IL-17 is yet to be elucidated. We sought a molecule that was induced by IFN-γ and known to inhibit IL-17: Indoleamine 2,3deoxygenase (IDO). It has been reported in several studies that IDO negatively regulates Th17 response in CIA models. Chen et al. demonstrated that CIA was ameliorated by IDO gene therapy through synovial IL-17 reduction and CD4+ cell apoptosis [22]. Criado et al. also reported that IDO deficient mice showed exacerbation of CIA with increased infiltration of Th17 cells in the inflammatory joints [23]. In our study, when the splenocytes of IFN-γ KO mice were treated with IFN-γ and 1-MT, the level of IL-17 was partially recovered compared to that of mice treated with IFN-γ alone. This was further supported by the experiments performed using IL-1Ra KO mice. When Th17 polarized cells were co-cultured with IFN-γ pretreated APCs from IFN-γ KO mice, the level of IL-17 in the supernatant was decreased compared to the co-culture with non IFN-γ pretreated APCs. This suggests that there may be a partial effect from the material secreted by the APCs, which was induced by IFN-γ. These findings suggest that enhancing IDO activity by IFN-γ treatment would be a promising therapeutic strategy in the treatment of RA. Although Schroecksnadel et al. [38] reported that IDO activity was already enhanced in the patients with RA, it seems that immunosuppressive activity of increased IDO is only partially able to suppress disease activity, and further stimulation of the enzyme can contribute to treatment.

However, the inhibitory function of IFN-γ could not be entirely explained by the action of IDO. In contrary to our expectations, IDO expression was not reduced in the IFN-γ KO mice (data not shown) even before CIA was induced. This may be due to the expression of IDO being induced by various stimuli other than IFN-γ. Moreover, IDO expression in the supernatant of co-culture of T cells and IFN-γ treated APCs was not assessed. Future studies are required to elucidate whether the level of IDO increases with IFN-γ treatment.

### Conclusion

We observed the exacerbation of CIA in IFN-γ KO C57BL/6 mice, confirming the protective role of IFN-γ in the development of CIA. This was abrogated in IFN-γ and IL-17 DKO mice, indicating that IFN-γ negatively regulates CIA by suppressing IL-17 function. The inhibitory function of IFN-γ on IL-17 production was abolished with IDO inhibitor, 1-MT, suggesting that IDO was involved in the inhibitory mechanism of IFN-γ.
